# Peptide Transporter CstA Imports Pyruvate in Escherichia coli K-12

**DOI:** 10.1128/JB.00771-17

**Published:** 2018-03-12

**Authors:** Soonkyu Hwang, Donghui Choe, Minseob Yoo, Sanghyuk Cho, Sun Chang Kim, Suhyung Cho, Byung-Kwan Cho

**Affiliations:** a Department of Biological Sciences and KI for the BioCentury, Korea Advanced Institute of Science and Technology, Daejeon, South Korea; b Intelligent Synthetic Biology Center, Daejeon, South Korea; Queen Mary University of London

**Keywords:** pyruvate, transporter, transposon sequencing, 3-fluoropyruvate, *Escherichia coli*

## Abstract

Pyruvate is an important intermediate of central carbon metabolism and connects a variety of metabolic pathways in Escherichia coli. Although the intracellular pyruvate concentration is dynamically altered and tightly balanced during cell growth, the pyruvate transport system remains unclear. Here, we identified a pyruvate transporter in E. coli using high-throughput transposon sequencing. The transposon mutant library (a total of 5 × 10^5^ mutants) was serially grown with a toxic pyruvate analog (3-fluoropyruvate [3FP]) to enrich for transposon mutants lacking pyruvate transport function. A total of 52 candidates were selected on the basis of a stringent enrichment level of transposon insertion frequency in response to 3FP treatment. Subsequently, their pyruvate transporter function was examined by conventional functional assays, such as those measuring growth inhibition by the toxic pyruvate analog and pyruvate uptake activity. The pyruvate transporter system comprises CstA and YbdD, which are known as a peptide transporter and a conserved protein, respectively, whose functions are associated with carbon starvation conditions. In addition to the presence of more than one endogenous pyruvate importer, it has been suggested that the E. coli genome encodes constitutive and inducible pyruvate transporters. Our results demonstrated that CstA and YbdD comprise the constitutive pyruvate transporter system in E. coli, which is consistent with the tentative genomic locus previously suggested and the functional relationship with the extracellular pyruvate sensing system. The identification of this pyruvate transporter system provides valuable genetic information for understanding the complex process of pyruvate metabolism in E. coli.

**IMPORTANCE** Pyruvate is an important metabolite as a central node in bacterial metabolism, and its intracellular levels are tightly regulated to maintain its functional roles in highly interconnected metabolic pathways. However, an understanding of the mechanism of how bacterial cells excrete and transport pyruvate remains elusive. Using high-throughput transposon sequencing followed by pyruvate uptake activity testing of the selected candidate genes, we found that a pyruvate transporter system comprising CstA and YbdD, currently annotated as a peptide transporter and a conserved protein, respectively, constitutively transports pyruvate. The identification of the physiological role of the pyruvate transporter system provides valuable genetic information for understanding the complex pyruvate metabolism in Escherichia coli.

## INTRODUCTION

Pyruvate is an important branch point intermediate of many metabolic pathways in living organisms. In central carbon metabolism, pyruvate is the final product of glycolysis and enters the tricarboxylic acid (TCA) cycle after the conversion to acetyl coenzyme A (acetyl-CoA) by pyruvate dehydrogenase. Additionally, pyruvate metabolism is highly interconnected with other metabolic pathways, including amino acid metabolism, fatty acid metabolism, and gluconeogenesis. When glucose is taken up at a high rate, the intracellular pyruvate concentration increases, resulting in a high ratio of pyruvate to phosphoenolpyruvate in Escherichia coli. This increased ratio activates the ArcA/B global two-component system, which participates in regulating a large number of genes in diverse biochemical reactions, including the repression of tricarboxylic acid cycle genes (1). As a central metabolic node, the intracellular pyruvate concentration is changed dynamically and must be tightly regulated (2). To control intracellular concentrations, pyruvate is also excreted into the medium in response to the dynamic cell status (3–5). For example, during the stationary phase, excreted pyruvate can be transported into the cell by carbon scavenging metabolism involving cyclic AMP (cAMP)/cAMP receptor protein regulation (4, 6).

Although metabolic fluxes connected to the pyruvate node have been examined under numerous conditions, the pyruvate transport system in E. coli remains unclear. The presence of at least two uptake systems and one excretion system for pyruvate transport was suggested using the mutant strains that grow with toxic pyruvate analogs (7). Interestingly, one uptake system constitutively imports pyruvate, while the other shows pyruvate uptake activity only when induced by pyruvate. Double mutants lacking both pyruvate-inducible and constitutive pyruvate transporters excreted intracellular pyruvate into the medium, indicating the existence of an independent pyruvate exporter. It has also been suggested that membrane protein YhjX function is related to changes in the extracellular pyruvate concentration (3). However, its mode of transport and substrate specificity remain unclear. Recently, BtsT (regulated by the BtsS/BtsR pyruvate sensing system) was identified as an inducible pyruvate/H^+^ symporter in E. coli (8). Despite these efforts, other pyruvate transporter genes in E. coli are yet to be identified.

Identifying microbial transporter genes is challenging for several reasons. Sequence homology-based computational approaches are often used to narrow down the putative transporter genes, which are then investigated by functional assays (9, 10). However, predicting substrate specificity based on sequence homology is often limited because of the flexible and broad substrate specificity of transporters (11, 12). In addition, most transporters are integrated with the cellular membrane, limiting the ability to perform biochemical assays to examine transport activity. To overcome these limitations, transposon sequencing (Tn-seq) is a robust and high-throughput method for genome-scale screening of transporter genes from a large mutant library (13–16). In this study, we employed the Tn-seq approach to identify the pyruvate transporter gene in E. coli. We serially cultured the Tn mutant library in the presence and absence of a toxic pyruvate analog to enrich transporter-deficient clones, followed by high-throughput sequencing of Tn insertion genome positions to determine the insertion frequency. The results identified 52 candidate genes, and further functional examination of the corresponding deletion strains revealed a putative pyruvate transporter that constitutively imports pyruvate. Along with the valuable genetic information for understanding the complex pyruvate metabolism in E. coli, our results provide a versatile pipeline for screening other transporter genes.

## RESULTS

### Clonal selection method from Tn insertion mutant library.

**FIG 1:**
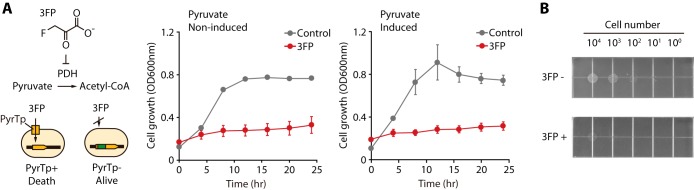
Growth inhibition tested with a toxic pyruvate analog. (A) Growth inhibition profile using toxic pyruvate analog 3FP (3-fluoropyruvate), under pyruvate-noninduced and -induced conditions. A final concentration of 1 mM 3FP was added at an OD_600_ of 0.1. PDH, pyruvate dehydrogenase; PyrTp, pyruvate transporter. (B) Colony spotting assay for E. coli K-12 MG1655 under growth inhibition by 3FP. The spot images of the plates were taken 32 h after spotting and incubation. 3FP−, M9 plus 0.2% sorbitol solid medium without adding 3FP; 3FP+, M9 plus 0.2% sorbitol solid medium with 1 mM 3FP added.

To select clones deficient in pyruvate transporter function in the Tn insertion mutant library, we first examined the toxic effect of the pyruvate analog 3-fluoropyruvate (3FP) on cell growth. In principle, 3FP is imported into cell through the pyruvate transporter and inhibits cell growth by binding to pyruvate dehydrogenase (7, 17–19). As expected, 3FP inhibited wild-type E. coli K-12 cell growth under pyruvate-induced and noninduced conditions (Fig. 1A). Additionally, the formation of wild-type colonies on M9 sorbitol medium was extremely retarded in the presence of 1 mM 3FP (Fig. 1B). Thus, clones deficient in pyruvate transporter function cannot uptake the toxic pyruvate analog and are enriched in the Tn insertion mutant library grown in M9 medium supplemented with the toxic pyruvate analog.

### Construction of Tn insertion mutant libraries and Tn-seq.

**FIG 2:**
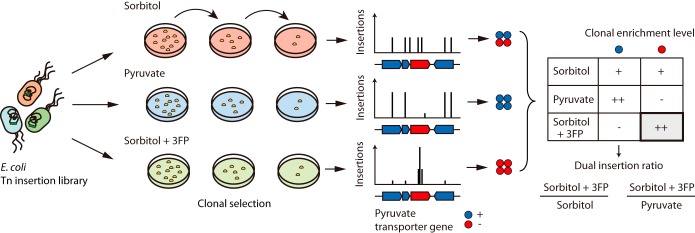
Transposon sequencing (Tn-seq) to identify pyruvate transporter genes in E. coli K-12 MG1655. Tn-seq strategy for the selection of differential clones. To construct the Tn mutant library, a transposon was inserted into a random site in the wild-type E. coli K-12 MG1655 genome. The library was serially grown on solid minimal medium with three different carbon sources: sorbitol, pyruvate, and sorbitol plus 1 mM 3FP. Through selection, clones with a detrimental insertion under each condition decreased in frequency. Blue circles indicate clones with the functional pyruvate transporter gene, while red circles indicate clones with transposon insertion within the pyruvate transporter gene. Reads obtained from high-throughput parallel sequencing were mapped to the reference genome. Candidate genes were selected considering the ratios of insertion frequencies between the sorbitol plus 3FP condition and the other two conditions.

**FIG 3:**
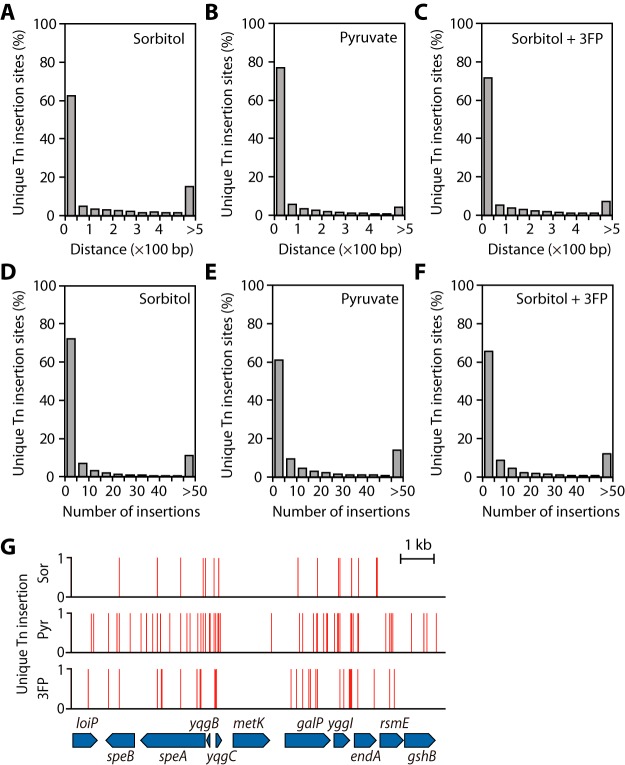
Distribution of unique transposon insertion sites across the genome. (A to C) The distributions of distances between transposon insertion sites across the genome under sorbitol (A), pyruvate (B), and sorbitol plus 3FP (C) conditions. (D to F) The distributions of insertion numbers at each insertion site in sorbitol (D), pyruvate (E), and sorbitol plus 3FP (F). (G) Example of a unique transposon insertion profile of an essential gene, *metK*.

One million viable Tn insertion mutants of E. coli K-12 strain MG1655, capable of growing on solid LB medium, were constructed, and 1 × 10^5^ colonies were selected from solid M9 medium supplemented with sorbitol, pyruvate, or sorbitol with 3FP (Fig. 2). Their unique Tn insertion positions and frequencies were examined using the Tn-seq approach (see Materials and Methods). A total of 3,623,464 reads were mapped with 18,967 unique Tn insertion sites under the sorbitol supplemented condition, 5,898,167 reads were mapped with 57,711 sites under pyruvate supplemented, and 3,680,158 reads were mapped with 36,830 sites under the sorbitol with 3FP supplemented condition. Thus, on average, the Tn-seq results yielded a unique Tn insertion per 245 bp under the sorbitol condition, per 80 bp under the pyruvate, and per 126 bp under the sorbitol with 3FP condition. To examine the distribution of these unique Tn insertion sites across the genome, distances between two adjacent Tn insertion sites were calculated, and their distribution is represented in Fig. 3A to C. Most of the pairs of adjacent unique Tn insertion sites were positioned within less than 50 bp, indicating their nearly uniform distribution across the genome. The instances of two Tn insertions >500 bp apart accounted for 15%, 4%, and 7% of the total insertion sites under sorbitol, pyruvate, and sorbitol with 3FP conditions, respectively. These results suggest the presence of essential genes (or enriched clones) under the respective growth conditions. Additionally, some Tn insertion sites had a higher insertion preference over the others, evidencing the existence of enriched clones (Fig. 3D to F). For instance, one essential gene, *metK*, encoding methionine adenosyltransferase, lacked Tn insertion in the protein-coding sequence, while the adjacent nonessential genes contained Tn insertions in significant portions of their coding regions (Fig. 3G). The GC contents of adjacent Tn insertion sites were 52 to 56%, showing good agreement with the results of a previous study (see Fig. S2 in the supplemental material) (20). Altogether, the Tn-seq results provided sufficient resolution for a comparative analysis of differential Tn insertion preferences under the respective growth conditions.

### Selection of pyruvate transporter gene candidates.

**FIG 4:**
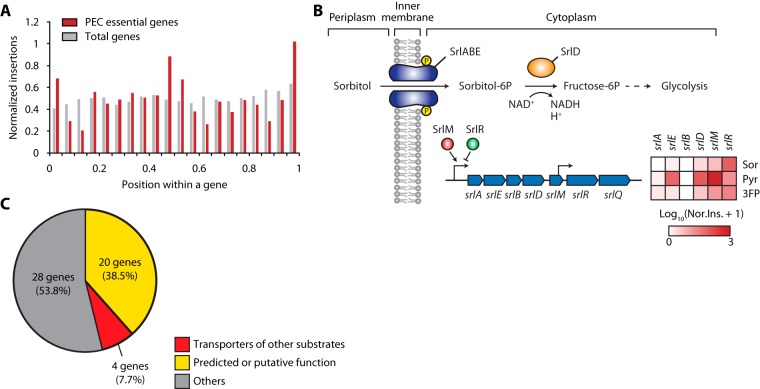
Candidate selection from Tn-seq results. (A) Distribution of normalized insertions over the relative position within profiling of E. coli chromosome (PEC) essential genes (*n* = 302) and total genes (*n* = 4,498). Insertions within 5% of the gene from the 3′ end were excluded from normalization for all genes. (B) Normalized insertion of genes involved in sorbitol metabolism. The pathway for sorbitol utilization and the normalized insertions in related genes depicted as a heat map. SrlM is a transcriptional activator and SrlR is a transcriptional repressor of the sorbitol metabolism operon. Sor, sorbitol; Pyr, pyruvate; 3FP, sorbitol plus 3FP condition; Nor.Ins., normalized insertion. (C) Three functional categories of the 52 selected candidate genes.

Transposons tend to be incorporated in some essential genes within significant portions of their 3′ ends. This insertion vulnerability enables the identification of nonessential protein segments (or functional domains) in the essential genes but may prevent the determination of gene essentiality under the conditions of interest. We observed similar insertion trends for essential genes in the Tn insertion libraries obtained under the growth conditions (Fig. 4A). To improve the essential gene calling from the Tn-seq results, the Tn insertion preference of each gene was represented as the sum of insertion counts within 95% of the region from the 5′ end of the gene normalized by gene length and total mapped reads under each condition. This enabled a comparison of the insertion preferences of all genes between the three conditions.

Genes with high normalized insertion values indicate an enrichment of Tn insertion clones of the corresponding genes. The gene is considered nonessential or beneficial for cell growth under the conditions when functionally deleted. In contrast, a low normalized insertion value for a gene indicates the elimination of Tn insertion clones of the corresponding gene, whose function is essential for cell growth under the conditions. We identified several genes showing differential normalized insertion values between the growth conditions supplemented by sorbitol, pyruvate, or sorbitol with 3FP as the carbon sources. For example, normalized insertion values of genes related to sorbitol metabolism, which are *srlD*, encoding sorbitol-6-phosphate 2-dehydrogenase, *srlA*, *srlB*, and *srlE*, encoding sorbitol-specific PTS enzyme components, and *srlM*, encoding a DNA-binding transcription activator, were higher under the pyruvate condition than under the sorbitol or sorbitol with 3FP condition (Fig. 4B; see also Table S1). As sorbitol was the sole carbon source under the sorbitol or sorbitol with 3FP condition, sorbitol uptake was found to be essential for cell growth as well as for the conversion to fructose 6-phosphate. However, the sorbitol metabolism pathway is nonessential under the pyruvate conditions. In the same manner, it was expected that genes with high normalized insertion values under sorbitol with 3FP conditions and low normalized insertion values under sorbitol or pyruvate conditions would demonstrate a high potential for pyruvate transporter function.

To identify potential pyruvate transporter genes, fold changes in the Tn insertions of 4,498 genes, which are the ratios of normalized insertion values under the sorbitol to those under the sorbitol with 3FP condition and the ratios of normalized insertion values under the pyruvate to those under the 3FP condition, were calculated (Table S1). The fold changes reflect changes in the frequencies of certain Tn insertion mutants according to pyruvate transporter functions and toxic pyruvate analog treatment. To exclude essential genes with low insertions under all three conditions, only genes with more than two unique insertions per 1 kb were selected. A total of 52 genes were selected by the criteria that fold changes were over 6-fold (see Table S2). Interestingly, 2 and 14 genes were located between 13 to 15.12 and 45.17 to 70 min, respectively, which were previously expected to be genomic regions of genes involved in constitutive and inducible pyruvate transporter functions (see Fig. S3) (7). Next, the functions of candidate genes were categorized into three groups as follows: group 1, predicted or putative inner membrane transporter genes such as *ybdD*, *yphA*, and *yohC*; group 2, transporter genes of other substrates such as *cstA* (peptide transporter gene), *dsdX* (serine transporter gene), and *mtr* (tryptophan transporter gene); and group 3, little relevant functions with transporters such as *bluR* (transcriptional repressor gene involved in the biofilm formation and acid resistance), *nac* (nitrogen assimilation regulon transcriptional regulator gene), and *moeB* (molybdopterin synthase sulfurylase gene) (Fig. 4C). Although most potential functional groups included genes related to predicted or putative inner membrane transporter function, genes in group 2 may also have pyruvate transporter function because of their broad substrate specificity and the multifunctional characteristics of transporters (12). Therefore, additional assays using deletion mutants of candidate genes were necessary to identify genes with pyruvate transport activity.

### Examination of pyruvate transporter activity of selected candidate genes.

**FIG 5:**
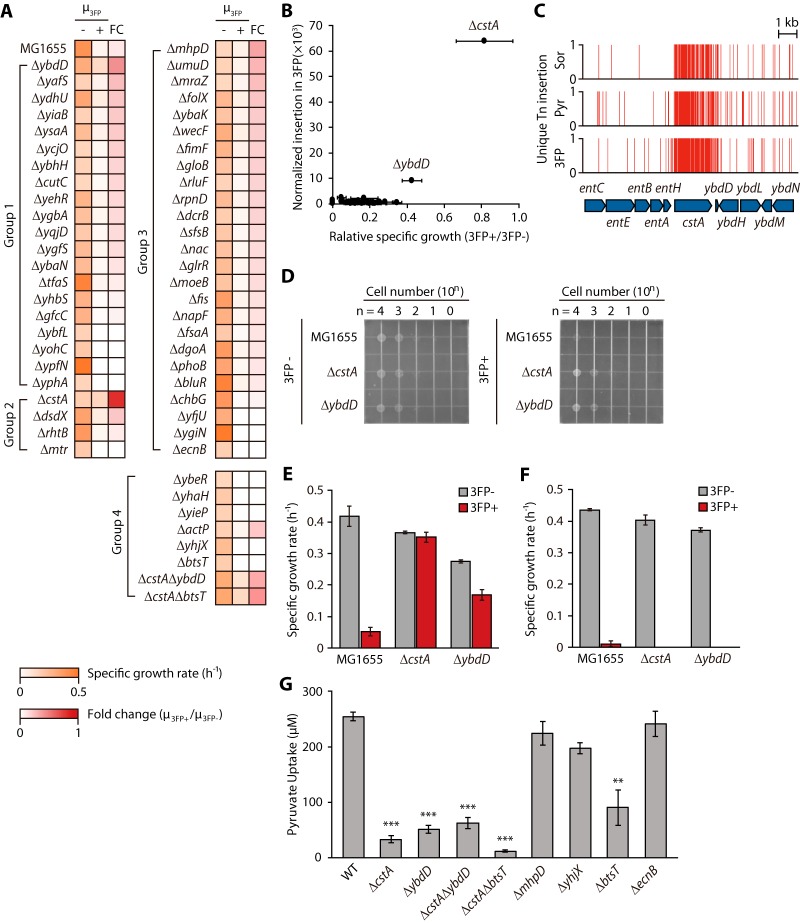
Final candidates, *cstA* and *ybdD*, selected from additional assays. (A) Specific growth rates of single gene knockout strains (from Keio collection, detailed in Materials and Methods) in sorbitol (3FP−) and sorbitol plus 3FP (3FP+) minimal medium. A total of 58 strains were tested, whose knockout genes were selected from Tn-seq. Group 1 genes belong to the “predicted or putative function” category, group 2 genes belong to the “transporters of other substrates” category, group 3 genes belong to the “other function” category, and group 4 includes additional genes without candidate groups from Tn-seq. (B) Correlation between normalized insertion values under the 3FP condition from Tn-seq and specific fold changes of growth rates from 3FP growth assays. *cstA* and *ybdD* showed strikingly high values in both experiments. (C) Unique transposon insertion profile of *cstA* and *ybdD*. Sor, sorbitol; Pyr, pyruvate; 3FP, sorbitol plus 3FP. (D) Colony spotting assay of E. coli K-12 MG1655, Δ*cstA*, and Δ*ybdD* strains under growth inhibition by 3FP. The spot images of plates were taken 32 h after spotting and incubation. 3FP−, M9 plus 0.2% sorbitol (solid) medium without adding 3FP; 3FP+, M9 plus 0.2% sorbitol (solid) medium with 1 mM 3FP added. (E and F) Growth inhibition of the three strains by 3FP under pyruvate-noninduced (E) and pyruvate-induced (F) conditions. 3FP−, minimal medium with sorbitol; 3FP+, minimal medium with sorbitol and 3FP. (G) Pyruvate uptake activity test by measuring the extracellular pyruvate concentrations 10 min after the pyruvate treatment of WT and mutant E. coli K-12 BW25113 strains. Statistical significance was calculated by Welch's *t* test from Python. ***, *P* < 0.001; **, *P* < 0.01.

To test the pyruvate uptake activity of the selected candidates, strains lacking the corresponding genes were obtained from the Keio strain collection (49 deletion strains; 3 strains were not available) (21). Each deletion strain was grown in M9 medium supplemented with sorbitol or sorbitol with 3FP to measure the inhibitory effect of 3FP on cell growth. The specific growth rates of the deletion strains in the functional categories were determined as fold changes (Fig. 5A and Table S2). Among them, the fold changes in the specific growth rates of Δ*cstA* and Δ*ybdD* strains were much greater than that of the wild-type strain, suggesting the involvement of these two genes in pyruvate transport. In addition to the two candidate genes, other potential genes (group 4) were also tested. Interestingly, the Δ*btsT* mutant lacking the inducible pyruvate transporter showed no growth in the medium supplemented with 3FP, thereby clearly indicating the existence of other pyruvate transporters for the uptake of 3FP. To examine the synergetic effects of these genes on pyruvate uptake, we additionally tested the growth inhibition of two double knockout strains (Δ*cstA* Δ*ybdD* and Δ*cstA* Δ*btsT* strains) by 3FP. No synergetic effect on the growth rates was observed in either the Δ*cstA* Δ*ybdD* or the Δ*cstA* Δ*btsT* mutant. Potential candidate genes with transport-related functions, such as *yhaH* and *yieP*, showed a less than 0.2-fold change in growth rates. In addition, transporter genes with potential substrate specificity toward pyruvate, such as *actP* and *yhjX*, were also tested, but cell growth was not observed under the sorbitol with 3FP condition (Table S2).

The fold changes in the specific growth rates of the deletion strains were weakly correlated with normalized insertion under 3FP conditions (Fig. 5B). Notably, the Δ*cstA* and Δ*ybdD* strains showed high fold changes in growth rates and were correlated with normalized insertion under 3FP conditions. The two genes showed clear differences in both Tn insertion frequency and unique Tn insertion sites under the three growth conditions (Fig. 5C). In addition, we examined the colony-spotting assays of Δ*cstA* and Δ*ybdD* strains (Fig. 5D). Three strains were normally grown on solid M9 medium supplemented with sorbitol, but colonies of Δ*cstA* and Δ*ybdD* strains were formed faster than those of the wild type on medium supplemented with sorbitol and 3FP. This result confirms that the Δ*cstA* and Δ*ybdD* strains avoid growth inhibition by 3FP.

The two genes are located at the genomic regions which were previously predicted as the genomic location of the constitutive pyruvate transporter (7). This transporter may be expressed constitutively, regardless of the presence of extracellular pyruvate. In contrast, the inducible pyruvate transporter is expressed only in the presence of extracellular pyruvate and is in a different genomic location (8). To test the induction of inducible pyruvate transporter by pyruvate for 3FP uptake, three strains were grown under pyruvate-induced conditions, with pregrowth in M9 medium supplemented by 0.2% pyruvate prior to growth in M9 medium supplemented with 0.2% sorbitol with and without 3FP. Additionally, the cells were grown under noninduced conditions directly in sorbitol media with and without 3FP. Under noninduced conditions, the fold changes in specific growth rates between the presence and absence of 3FP were 0.13 for the wild-type strain, 0.96 for the Δ*cstA* strain, and 0.61 for the Δ*ybdD* strain, which were consistent with the results from the growth inhibition assay (Fig. 5E). However, under pyruvate-induced conditions, the fold changes were 0.02 for the wild-type strain and zero for the Δ*cstA* and Δ*ybdD* strains, which showed complete growth inhibition by 3FP (Fig. 5F). Because the inducible pyruvate transporter is expected to import 3FP under pyruvate-induced conditions, both the Δ*cstA* and Δ*ybdD* strains showed reduced growth rates. These results confirmed that *cstA* and *ybdD* are constitutive pyruvate transporter genes.

In addition to the growth assay using 3FP, the pyruvate uptake activity of the deletion strains was investigated by measuring the extracellular pyruvate concentrations under the pyruvate-induced condition (Fig. 5G). Among the eight knockout strains, five strains (Δ*cstA*, Δ*ybdD*, Δ*btsT*, Δ*cstA* Δ*ybdD*, and Δ*cstA* Δ*btsT* strains) imported significantly less pyruvate than the wild-type strain (*P* < 0.01, Welch's *t* test). Pyruvate uptake levels of the mutant strains were reduced to 13%, 20%, 36%, 25%, and 5% of the wild-type strain, respectively, thereby indicating that both *cstA* and *ybdD* are required for constitutive uptake of pyruvate. In addition, the inducible pyruvate transporter BtsT also imports pyruvate in the presence of 0.1 to 0.5 mM extracellular pyruvate (8). Notably, the double knockout mutant (Δ*cstA* Δ*btsT*), which was expected to be deficient in both inducible and constitutive pyruvate transport systems, showed the lowest fold change in pyruvate uptake (0.05). This result is considered to be a cumulative effect of both the genes on pyruvate transport. Another knockout strain, the Δ*yhjX* mutant lacking a putative transporter gene, showed a slightly reduced fold change in pyruvate uptake (0.77), indicating YhjX is potentially involved in the formation of a different pyruvate uptake system. In the 3FP growth inhibition assay, a Δ*mhpD* mutant strain showed the third highest fold change in growth rate; however, the strain showed no significant change in pyruvate uptake (*P* > 0.01, Welch's *t* test) (Fig. 5G). The *mhpD* gene encodes 2-keto-4-pentenoate hydratase, involved in aromatic compound metabolism (22). The final metabolic products from this pathway are pyruvate and acetyl-CoA, which are eventually converted to succinate through the TCA cycle. Thus, the cellular function of MhpD and the absence of pyruvate uptake activity in its mutant strain suggest that the change in the growth rate of the Δ*mhpD* mutant could be due to the metabolic perturbation caused by gene deletion. As a negative control, the Δ*ecnB* strain, whose knocked out gene encoded the entericidin B membrane lipoprotein and showed no growth under the 3FP condition, showed an uptake level closely similar to that of the wild-type strain. These results support the impairment of pyruvate uptake activities in the Δ*cstA* and Δ*ybdD* strains. The deletion strains incubated without pyruvate treatment showed negligible or no pyruvate uptake, indicating no effect of pyruvate export function on the extracellular measurement of pyruvate levels.

Next, we examined the sequence homology of CstA against other related proteins in other bacterial species. CstA showed high similarity (>90%) with carbon starvation protein A, which is highly conserved in other bacterial species. A multiple-sequence alignment with pyruvate transporter genes in other species showed no significant sequence homology between CstA and other transporters or between other transporters themselves. Only BtsT, which is identified as an inducible pyruvate transporter in E. coli, showed significant similarity (60%). Altogether, the results from the growth inhibition assays using 3FP treatment and the pyruvate uptake activity test verified that the constitutive pyruvate transporter system consisted of *cstA* and *ybdD* in E. coli.

## DISCUSSION

In this study, we identified genes encoding pyruvate transport activity in E. coli. Pyruvate is a dynamically regulated metabolite positioned at an important node that interconnects diverse catabolic and biosynthetic pathways. In response to the metabolic states in the cell, pyruvate can be excreted into the extracellular medium, whereas it can also be taken up again into the cell under certain conditions, such as carbon starvation (3). This dynamic and reversible regulation of intracellular and extracellular pyruvate concentrations suggests that the pyruvate transport system maintains homeostasis in E. coli. Additionally, more than four genes related to pyruvate transport in E. coli have been identified: an inducible transporter BtsT, a constitutive transporter, a putative inducible transporter YhjX, and an unknown exporter (3, 7, 8, 17). Despite its importance in bacterial metabolism, only one pyruvate transporter gene, encoding the inducible transporter, has been identified and verified in E. coli.

Indeed, Tn mutants lacking pyruvate transporter function were enriched in the presence of 3FP, whereas supplementation with sorbitol or pyruvate provided nontoxic carbon sources for normal cell growth. The fold change in the Tn insertion frequency of the enriched mutant libraries under the three growth conditions revealed 52 candidates for examination of their pyruvate transporter function. These gene identification strategies are valid and effective for three reasons. (i) A number of the transporters have a broad substrate specificity, or the other transporters can replace the transporter function of interest (12). On the other hand, Tn-seq reveals quantitative differences in growth fitness levels between clones with diverse degrees of transporter function. (ii) A gene of interest may be multifunctional or misannotated because it exhibits different functions than expected (23, 24). Thus, sequence homology-based target selection for functional assays may result in false-positive calling. In contrast, the Tn-seq library contains entire gene set that can reduce bias. (iii) Our results filled the gap between the genetic information and phenotypic characteristics of the pyruvate transporter gene, which transports an important and abundant metabolite in E. coli. The genetic information obtained from this study provides a basis for understanding the tight regulation of intracellular pyruvate concentrations.

The final candidate genes, *cstA* and *ybdD*, are known as genes for a peptide transporter induced by carbon starvation and a conserved protein, respectively. Thus, our study is the first report demonstrating their pyruvate transport function. CstA is an inner membrane protein consisting of 701 amino acids and is regulated by the cAMP-cAMP receptor protein complex at the transcriptional level under carbon starvation conditions (25, 26). CstA is a highly conserved protein among several bacterial species, and its peptide transport function has been examined in E. coli and Campylobacter jejuni (23, 25). In addition, CstA is related to other functions, such as biofilm formation, motility, and agglutination (23, 24). It is known that the carbon storage regulator CsrA negatively regulates the expression of CstA at the translational level (26). Therefore, the pyruvate uptake level of the CsrA knockout mutant was expected to be higher than that of the wild type, but the knockout strain was found to be growth defective instead. YbdD is a small protein consisting of 65 amino acids and whose function is currently unknown. The genomic location of the gene encoding this protein is immediately next to *cstA*, indicating the potential for a protein-protein interaction with CstA to transport pyruvate. For instance, some small membrane protein genes are located in close vicinity to membrane transporters, such as *mctB* and *mctC* in Corynebacterium glutamicum, as well as *yjcH* and *actP* in E. coli (11). In Bacillus subtilis, PftAB is the recently identified inducible pyruvate transporter, also encoded by two adjacent genes (27). To demonstrate the role of YbdD in the pyruvate transport system, the direct interaction between CstA and YbdD should be further assessed using a certain assay, such as a bacterial two-hybrid system (28, 29).

In addition to the putative pyruvate-sensing two-component system YpdA/YpdB and its target YhjX, it has been suggested that the histidine kinase/response regulator system consisting of BtsS/BtsR and its target BtsT plays an important role in the cellular response to extracellular pyruvate as well as carbon starvation conditions (30, 31). The BtsS/BtsR system is induced by high concentrations of Casamino Acids or pyruvate with high binding affinity (30). Recently, its target, BtsT, was identified as a specific pyruvate transporter induced by extracellular pyruvate (8). The high sequence similarity between CstA and BtsT (approximately 60%) indicates the possibility that both proteins have similar functions, namely, the transport of peptides as well as pyruvate. The additive effect of *cstA*, *ybdD*, and *btsT* deletions on pyruvate uptake was also tested. Although YbdD is a small protein with an unknown function, its single knockout strain showed levels of 3FP-induced growth inhibition and pyruvate uptake similar to those of a *cstA* single knockout strain, thus implying that YbdD is essential for the pyruvate transport system. The Δ*cstA* Δ*ybdD* double knockout mutant showed no significant difference in either growth rate or pyruvate uptake compared to those of the respective single knockout mutants, indicating that the two proteins constitute a single pyruvate transporter complex. In contrast, the strain with double knockout of both pyruvate transport systems, the Δ*cstA* Δ*btsT* mutant, showed an additive effect on pyruvate uptake. A triple knockout mutant, the Δ*cstA* Δ*ybdD* Δ*btsT* strain, was also constructed, but it did not grow under the growth conditions tested, hence indicating that those pyruvate uptake systems are essential for cell growth. Knockout strains lacking the other candidate genes were also examined. Among those, the Δ*mhpD* strain, lacking the gene encoding 2-keto-4-pentenoate hydratase, showed the third highest change of growth rate. MhpD is associated with pyruvate metabolism; the final metabolic products are pyruvate and acetyl-CoA, which are eventually converted to succinate through the TCA cycle (22). However, the strain showed no significant change in pyruvate uptake, which could be due to its metabolic perturbation. The Δ*yhjX* mutant, whose knockout gene is downstream of the genes encoding the YpdA/B pyruvate-sensing two-component system, showed no change in growth rate with slightly reduced uptake, hence considered to be possibly related to the pyruvate uptake system.

**FIG 6:**
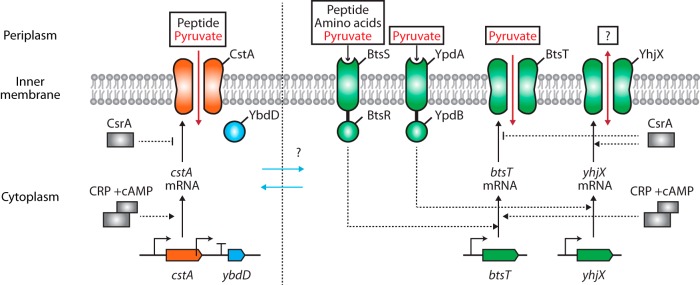
Schematic diagram of CstA constitutive pyruvate transporter system and two pyruvate-sensing systems. Rectangles indicate cAMP receptor protein (CRP) or CsrA regulators. Black arrows indicate the transcription and expression of the gene as protein. Dashed lines with arrowheads mean activation, while those with line ends mean repression of the process. Red arrows indicate the transport of substrates, and blue arrows indicate putative interactions between previously studied constitutive and inducible pyruvate transport systems.

These studies indicate that there are tightly and dynamically regulated systems for sensing and transporting extracellular pyruvate, peptides, and amino acids according to the growth phases related to overflow metabolism. Because amino acids can be metabolized to pyruvate as a resource for various metabolic pathways, we expect that peptide utilization and the pyruvate concentration controlled by transporters are closely related. Thus, our finding that the peptide transporter CstA imports pyruvate may be the missing link in the pyruvate and peptide/amino acid-sensing network in E. coli (Fig. 6).

On the basis of the collective analyses of Tn-seq, the 3FP growth inhibition assay, and the pyruvate uptake assay, we concluded that CstA and YbdD import pyruvate constitutively. However, the reduced growth inhibition by 3FP might have resulted either from metabolic perturbation induced by the knockout or from regulatory cross talk between CstA/YbdD and pyruvate transporters. The reduced pyruvate uptake could also be due to the indirect feedback effect; for example, peptides imported by CstA may activate the expression of other pyruvate transporters for the uptake of pyruvate. Thus, further investigation of the mode of action and broad substrate specificity of CstA is needed, along with an understanding of the relationship between carbon starvation and constitutive pyruvate uptake activity. The multifunctional properties of CstA also remain unclear. One limitation of our study is that only the constitutive pyruvate transporter system was identified and not the genes encoding inducible pyruvate transporters. This is because the selection conditions for transposon mutant enrichment were pyruvate-noninduced conditions. Therefore, the inducible pyruvate transporter genes, including *btsT*, can be identified using a Tn mutant library constructed from cells lacking *cstA* under pyruvate-induced conditions. Additionally, we can reconstruct the pyruvate-sensing and transport network to understand complex pyruvate metabolism in E. coli.

## MATERIALS AND METHODS

### Bacterial strains and growth conditions.

Bacterial strains used in this study are listed in Table S3 in the supplemental material. Wild-type E. coli K-12 MG1655 cells were grown in 3 ml of Luria-Bertani (LB) medium at 37°C. The cells were transferred and grown in M9 medium (0.493 g/liter MgSO_4_ · 7H_2_O, 0.015 g/liter CaCl_2_ · 2H_2_O, 12.8 g/liter Na_2_HPO_4_ · 7H_2_O, 3 g/liter KH_2_PO_4_, 0.5 g/liter NaCl, and 5 g/liter NH_4_Cl) supplemented with 0.2% of a carbon source, which was d-sorbitol (Sigma-Aldrich, St. Louis, MO) or sodium pyruvate (Sigma-Aldrich). Escherichia coli K-12 strain BW25113 and the Keio knockout collection were purchased from Coli Genetic Stock Center (New Haven, CT) and Dharmacon (Lafayette, CO), respectively, and used in growth and uptake tests (21).

### Growth inhibition test by toxic pyruvate analog.

Cells were grown in 3 ml of LB medium for 8 h at 37°C. For pyruvate-noninduced, pyruvate-induced, and control growth conditions (7), the cells were washed twice with 1 ml of M9 medium supplemented with 0.2% sorbitol, 0.2% pyruvate, and 0.2% sorbitol, and then inoculated in 30 ml of M9 medium supplemented with 0.2% sorbitol, 0.2% pyruvate, and 0.2% sorbitol to reach an optical density at 600 nm (OD_600_) of 0.05. For pyruvate-noninduced growth, the cells were grown in M9 medium supplemented with 0.2% sorbitol for one generation. In contrast, for pyruvate-induced growth, the cells were grown overnight in M9 medium supplemented with 0.2% pyruvate and then transferred to and grown in M9 medium supplemented with 0.2% sorbitol for one generation. After one generation of growth, 400 μl of the culture was transferred to a 48-well plate (SPL Life Sciences Co., Ltd., Pocheon, South Korea). Next, β-fluoropyruvic acid sodium salt monohydrate (3FP) (Sigma-Aldrich) was added to the culture at a final concentration of 1 mM, and the culture was incubated 37°C in a Synergy H1 microwell reader (BioTek, Winooski, VT) with double-orbital continuous shaking at a frequency of 2 mm. Every 15 min, the OD_600_ of each well was measured. For the growth inhibition assay, two deletion strains (Δ*cstA* and Δ*ybdD*) were grown under both pyruvate-noninduced and pyruvate-induced conditions as described above and washed twice with 1 ml of M9 medium supplemented with 0.2% sorbitol. The washed cells were inoculated in 50 ml of the same medium and incubated at 37°C in a shaking incubator at 220 rpm.

### Colony spotting assay.

Cells were grown in 3 ml of LB medium at 37°C overnight and serially diluted in M9 medium containing 0.2% sorbitol from 1 × 10^9^ to 1 × 10^2^ cells as duplicates. Next, 10 μl of the five most diluted cultures were spotted onto solid M9 medium with 0.2% sorbitol with and without 1 mM 3FP. The culture plates were imaged in the Gel Doc XR+ (Bio-Rad, Hercules, CA) every 1 h. The images were obtained using Image Lab software.

### Clonal selection from Tn mutant library.

Escherichia coli K-12 MG1655 cells were treated with EZ-Tn*5* <KAN-2>Tnp transposomes (Epicentre, Madison, WI) to construct the Tn mutant library (32). After overnight growth in LB medium, three samples of 2 × 10^9^ cells were collected, washed twice with M9 salt solution, and spread on solid M9 medium supplemented with 0.2% sorbitol, 0.2% pyruvate, and 0.2% sorbitol with 1 mM 3FP. Although pyruvate supplementation results in the highest induction of pyruvate uptake activity, pyruvate transporter-deficient clones are either unable to grow or show slow growth in M9 medium supplemented with pyruvate as the sole source of carbon (7). In contrast, pyruvate transporter-deficient clones grow normally under sorbitol or sorbitol with 3FP supplemented conditions. Sorbitol partially induces pyruvate uptake activity with a relatively low level of catabolite repression compared to that by glucose (7). For clones with intact pyruvate transporter, normal growth can be expected under both pyruvate and sorbitol conditions. Thus, after three serial passages under the given growth conditions, we expected that pyruvate transporter-deficient clones would be outcompeted and enriched under sorbitol with 3FP conditions compared to that under sorbitol or pyruvate conditions. Therefore, after 36 h of incubation, approximately 5 × 10^6^ colonies were collected from each plate. To enrich outcompeted clones under each condition, we repeated the clonal selection steps for three passages. The colonies were observed with a phase-contrast microscope to detect contamination during incubation.

### Tn-seq.

The collected cells were divided into 100 aliquots to extract their genomic DNA and generate the Tn-seq library by the modified 3-step PCR method (33). Primers used in this study are listed in Table S4. The cell pellet was lysed by adding 600 μl of nuclei lysis solution (Promega, Madison, WI) and incubating at 80°C for 10 min. Next, 12 μg of RNase A (Qiagen, Hilden, Germany) was added and the mixture was incubated at 37°C for 30 min to degrade the RNA. Next, 200 μl of protein precipitation solution (Promega) was added to precipitate the proteins. The sample was centrifuged at 17,000 × *g* for 3 min, and 600 μl of supernatant was mixed with an equal volume of isopropanol to precipitate genomic DNA. The genomic DNA was collected as a pellet by centrifugation and then washed twice with 600 μl of 70% ethanol to remove remaining salts. After drying, the genomic DNA was dissolved in 50 μl of nuclease-free water. Ten of the extracted genomic DNA samples were mixed at the same concentrations (100 ng/μl) and used as the template for subsequent PCR (Fig. S1). In the first PCR step, one primer specifically anneals to the Tn sequence, while the other primer anneals to a random genomic region next to the Tn junction. The random primer contains 8 nucleotides and one of four 5-bp sequences (GCTGG, CCAGC, CTGGC, and TGGCG). These 3′-end 5-bp sequences of the random primer appear approximately once per 51 bp in the whole genome. In the second and third PCR steps, sequencing adapters were added to both ends for Illumina sequencing. Each library representing different selection conditions contained different sequencing indexes of the 3′-end adapter. The first PCR mixture (10 μl) contained 2× HotStarTaq master mix (Qiagen), 0.1 μl of pFU-X (Solgent, Daejeon, South Korea), 1 μl of the genomic DNA sample, and 0.5 μM each primer (Fig. S1). The PCR mixtures were cycled at 95°C for 5 min (one cycle), 94°C for 20 s, 63°C for 45 s, and 72°C for 3 min (total 9 cycles), 94°C for 20 s, 63°C for 45 s, and 72°C for 3 min (total 12 cycles), 94°C for 20 s, 44°C for 45 s, 72°C for 3 min, and 72°C for 7 min on a C1000 thermal cycler (Bio-Rad). The first PCR products were diluted by 100-fold and used as the templates for the second PCR with primers (Table S4). The PCR mixtures were cycled at 95°C for 5 min (one cycle), 94°C for 20 s, 63°C for 45 s, 72°C for 5 min, 94°C for 20 s, 63°C for 45 s, 72°C for 5 min, 94°C for 20 s, 53°C for 45 s, and 72°C for 5 min (9 cycles), 94°C for 20 s, 63°C for 45 s, 72°C for 5 min, 94°C for 20 s, 53°C for 45 s, and 72°C for 5 min (8 cycles), and 72°C for 7 min. The second PCR products were diluted by 100-fold and used as the templates for the third PCR with primers (Table S4). The PCR mixtures were cycled under same conditions as the second PCR. The third PCR products were pooled and loaded onto a 1% LE agarose gel. After running at 100 V for 50 min, 300- to 700-bp bands were excised from the gel and extracted with the MinElute gel extraction kit (Qiagen). The concentration of each library was measured with the Qubit dsDNA HS assay kit (Thermo Fisher Scientific, Waltham, MA). The quality of each library was checked on a 2% SYBR gold gel and with a Kapa library quantification kit (Kapa Biosystems, Wilmington, MA). Tn-seq libraries were then sequenced using an Illumina MiSeq v2 instrument with MiSeq reagent v3 kits (150 cycled single-end reads) according to the manufacturer's protocol (Illumina, San Diego, CA).

### Sequencing data analysis.

Sequencing data were processed using CLC Genomics Workbench 6.5.1 software (CLC Bio, Aarhus, Denmark). Low-quality reads with more than two ambiguous nucleotides and sequencing reads to the PhiX genome (NC_001422) were discarded. To obtain only Tn-gDNA junction sequences, the 5′-end Tn sequences were trimmed. Trimmed reads were mapped to the reference genome (NC_000913.3). Detailed parameters are shown in Fig. S1. Mapped read information was exported in a BAM file format, which was further converted to a GFF file format containing Tn insertion counts for each genomic position. The GFF file was visualized on SignalMap (v2.0.0.5; Roche NimbleGen, Basel, Switzerland). To reduce selection errors, only genes with 2 or more Tn insertion sites were analyzed. For each of the remaining genes, the Tn insertion frequency was calculated by using the sum of insertion counts within 95% of the gene from the 5′ end. This value was normalized by gene length and total mapped reads under each condition.

### Growth inhibition assay of Keio strains.

Keio strain cells were inoculated in 3 ml of LB medium supplemented with 25 μg/ml kanamycin. Cells were allowed to grow overnight and then washed twice with 1 ml of M9 medium supplemented with 0.2% sorbitol and inoculated in 50 ml of M9 medium supplemented with 0.2% sorbitol to an OD_600_ of 0.05. After one generation of growth, 100 μl of the culture was transferred to a 96-well plate (SPL Life Sciences Co., Ltd.). Next, 3FP was added to the culture at a final concentration of 1 mM and the culture was incubated 37°C in a Synergy H1 microwell reader with double-orbital continuous shaking at a 2-mm frequency. Every 30 min, the OD_600_ of each well was measured.

### Construction of knockout strains.

Two double knockout strains (Δ*cstA* Δ*ybdD* and Δ*cstA* Δ*btsT* strains) and one triple knockout strain (Δ*cstA* Δ*ybdD* Δ*btsT* strain) were constructed by lambda recombination (34). Primer sequences used in the knockout constructions are listed in Table S4. Briefly, genomic copies of the 5′ coding region of *cstA* to the 3′ coding region of *ybdD* (cstA_KO_F and ybdD_KO_R) or the coding region of *btsT* (yjiY_KO_F and yjiY_KO_R) were deleted with kanamycin-resistant DNA cassettes amplified from pKD13 in pKD46 carried by strain BW25113. To produce the respective Δ*cstA* and Δ*btsT* mutants, pCP20 (34) was introduced into the strains before the recombination to remove the kanamycin cassette flanked by FLP recognition sites. pKD46 and pCP20 were cured by an overnight incubation at 42°C.

### Measurement of pyruvate uptake activity.

We measured extracellular pyruvate concentrations after pyruvate treatment (7, 17). The cells were grown in 3 ml of LB medium for 5 h at 37°C in a shaking incubator at 220 rpm. The cells were washed with 1 ml of M9 medium supplemented with 0.2% sorbitol twice and inoculated in 50 ml of M9 medium supplemented with 0.2% sorbitol in biological triplicates. A total of 10 ml of cultures at exponential growth phase was collected and washed twice with 10 ml of ice-cold 1% NaCl. The cells were then resuspended in 10 ml of M9 medium without any carbon sources. To examine pyruvate uptake activity, 300 μM sodium pyruvate was added to each sample. After 10 min of incubation at room temperature, the cells were centrifuged at 3,134 × *g* for 5 min at 4°C. The supernatants were collected and stored at −20°C until further analysis. Extracellular pyruvate concentrations were determined with the Cedex Bio HT Analyzer (Roche, CustomBiotech) according to the manufacturer's instructions.

### Sequence analysis.

Transmembrane domains and conserved domains were predicted by TMHMM and Pfams, respectively. A multiple alignment of related transporter sequences was conducted by Geneious software (Auckland, New Zealand) with global alignment and a blosum62 matrix (midrange). Phylogenetic trees were drawn by MEGA 7 software with ClustalW, blosum62 matrix, and maximum likelihood statistical methods.

## Supplementary Material

Supplemental material
