# Supplementary material for: Peptide Transporter CstA Imports Pyruvate in Escherichia coli K-12
Source: J Bacteriol. 2018 Mar 12;200(7):e00771-17. doi: 10.1128/JB.00771-17 (PMC5847655; doi:10.1128/JB.00771-17)
Supplement: Supplemental material [file supp_200_7_e00771-17__index.html]

Supplemental material 

# Peptide Transporter CstA Imports Pyruvate in Escherichia coli K-12

## Supplemental material

- Supplemental file 1 -

  Fig. S1 (Tn-seq scheme: library construction and mapping), S2 (ATGC ratio of bases adjacent to transposon insertions), and S3 (Genomic location of candidate genes selected from Tn-seq) and Tables S2 (Specific growth rate of 52 candidates and others), S3 (Bacterial strains used), and S4 (Primers used)

  PDF, 654K
- Supplemental file 2 -

  Table S1 (Normalized insertion of total 4,498 genes of *E. coli* K-12 MG1655)

  XLSX, 484K
