# Supplementary material for: Peptide Transporter CstA Imports Pyruvate in Escherichia coli K-12
Source: J Bacteriol. 2018 Mar 12;200(7):e00771-17. doi: 10.1128/JB.00771-17 (PMC5847655; doi:10.1128/JB.00771-17)
Supplement: Supplemental material [file JB.00771-17_zjb999094681s1.pdf]

Supplemental Information

Peptide transporter CstA imports pyruvate in *Escherichia coli* K-12

Soonkyu Hwang[1], Donghui Choe[1], Minseob Yoo[1], Sanghyuk Cho[1], Sun Chang Kim[1,2],

Suhyung Cho[1], and Byung-Kwan Cho[1,2,*]

Figure S1. Tn-seq scheme: library construction and mapping.

Figure S2. ATGC ratio of bases adjacent to transposon insertions.

Figure S3. Genomic location of candidate genes selected from Tn-seq

Table S1. Normalized insertion of total 4498 genes of *E. coli* K-12 MG1655.

Table S2. Specific growth rate of 52 candidates and others.

Table S3. Bacterial strains used in this study.

Table S4. Primers used in this study.

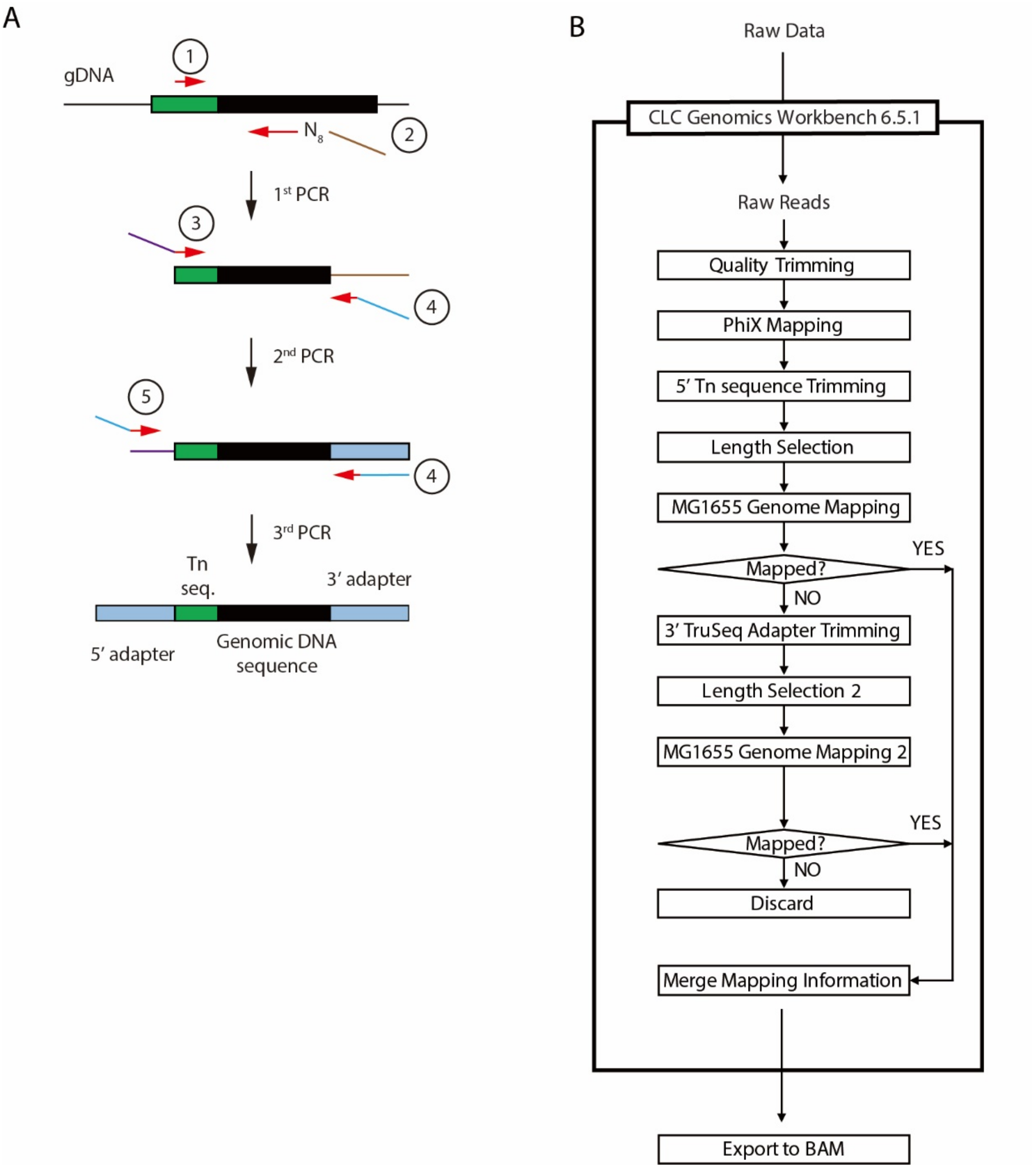

Figure S1. Tn-seq process scheme. (A) Overview of 3-step PCR method. Black thin line represents the genome, whereas green bar is an inserted transposon sequence and black bar is a genomic DNA sequence next to the transposon-inserted site. White blue bars are sequencing adapters for Illumina sequencing. Red arrows are primers, whose names and sequence information are shown in **Table S4**. Eight of ‘N’ indicates random sequences for random annealing. Sequences highlighted by white blue color are indices for random primers and adapter primers. (B) Data processing and mapping workflow of raw reads from Tn-seq. Quality trimming: quality score was 0.05, and reads with more than two ambiguous nucleotides were discarded. 5′ Tn5 sequence trimming: reads with Tn5 sequence were collected, while others were discarded. 5′ Tn5 sequence was trimmed with the following settings: minimal internal score 10 and end score was not allowed. Length selection: reads less than 15 bp in length were discarded. *Escherichia coli* K-12 MG1655 genome mapping: reads were mapped to *E. coli* K-12 MG1655 reference genome (NC_000913) with the following parameters: similarity score 0.9, length fraction 0.9, non-global, and ignore for multiply mapped reads. 3′ Truseq adapter trimming: Among unmapped reads, reads with part

of the 3′ Truseq adapter sequence were collected. Next, 3′ adapter sequence was trimmed with the following settings: minimal internal score 10 and end score 3. *Escherichia coli* K-12 MG1655 Genome mapping 2: remaining reads were mapped to *E. coli* K-12 MG1655 reference genome (NC_000913) with the followed parameters: similarity score 0.9, length fraction 0.8, non-global, and ignore for multiply mapped reads. All mapped reads were merged and converted to BAM format.

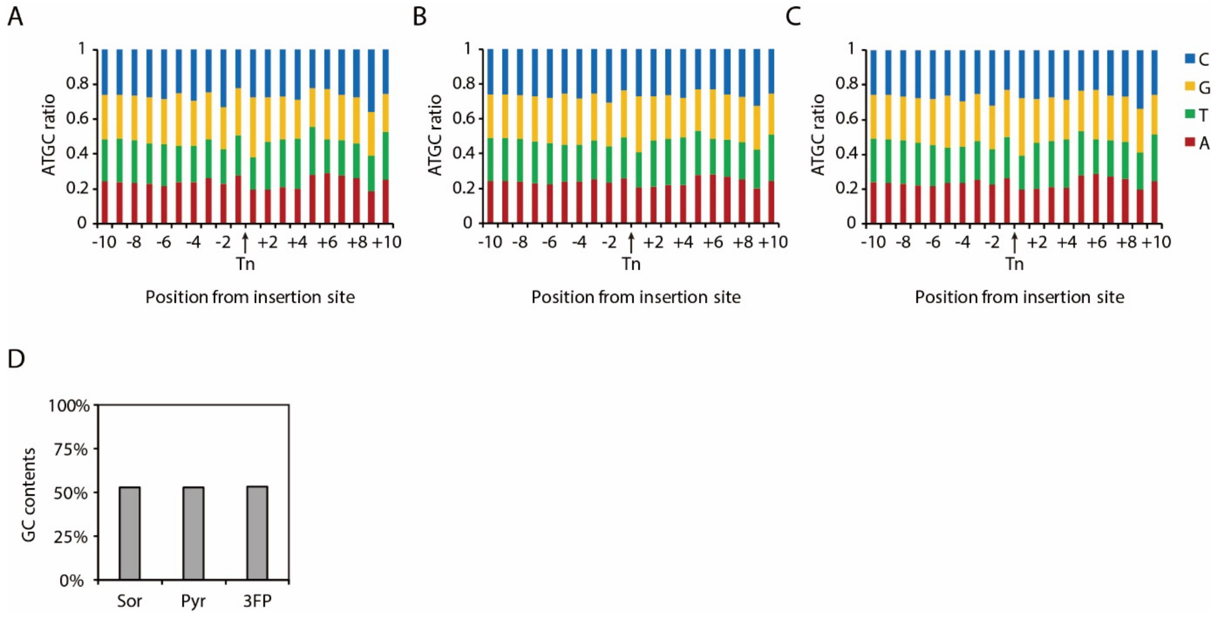

Figure S2. ATGC ratio of bases adjacent to transposon insertions. (A–C) ATGC ratio of 10 bp upstream and downstream of transposon insertions. The ratio patterns of sorbitol (A), pyruvate (B), and sorbitol + 3FP (C) are illustrated. (D) GC contents of sequences near transposon insertions. Sequences 10 bp upstream and downstream of transposon insertion sites were analyzed.

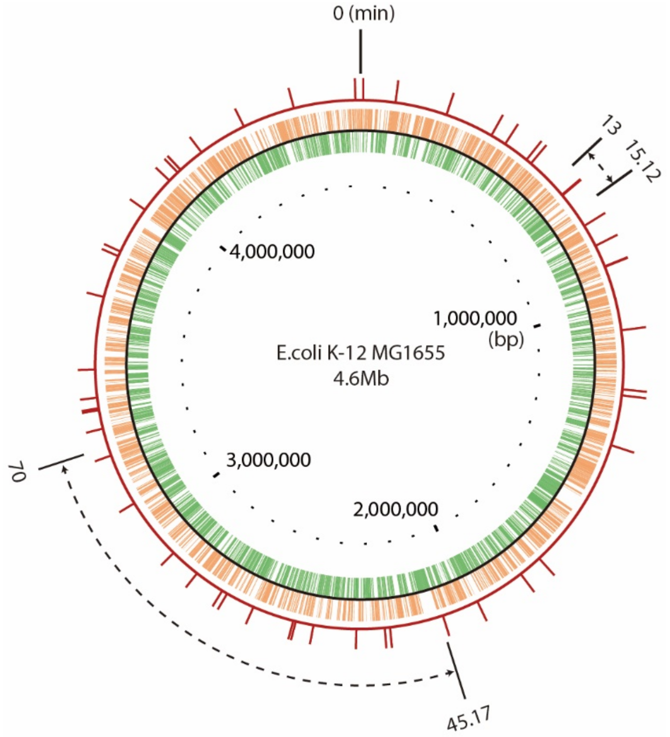

Figure S3. Genomic location of candidate genes selected from Tn-seq. Red lines are locations of candidate genes, yellow and green lines are locations of all genes of wild-type, and innermost black dotted circle shows genomic position (base pairs). Black lines and outermost dotted lines show genomic position (min).

Table S1. Normalized insertion of total 4498 genes of *E. coli* K-12 MG1655.

Available online.

Table S2. Specific growth rate of 52 candidates and others.

Group[a]	Gene	Function	Specific growth rate μ (h^{-1})		
			3FP -	3FP +	FC
I	*ybdD*	DUF466 family protein	0.311	0.132	0.425
	yafS	Putative S-adenosyl-L-methionine-dependent methyltransferase	0.169	0.043	0.255
	ydhU	Putative cytochrome b subunit of YdhYVWXUT oxidoreductase complex	0.330	0.073	0.220
	yiaB	YiaAB family inner membrane protein	0.174	0.036	0.209
	ysaA	Putative hydrogenase, 4Fe-4S ferredoxin-type component	0.248	0.051	0.205
	ycjO	Putative sugar ABC transporter permease	0.157	0.029	0.188
	ybhH	Putative PrpF family isomerase	0.166	0.027	0.164
	cutC	Copper homeostasis protein	0.154	0.024	0.158
	yehR	Lipoprotein, DUF1307 family	0.278	0.044	0.157
	ygbA	Uncharacterized protein	0.231	0.034	0.149
	yqjD	Putative membrane-anchored ribosome-binding protein	0.200	0.028	0.138
	ygfS	Putative 4Fe-4S ferredoxin-type oxidoreductase subunit	0.252	0.034	0.137
	ybaN	DUF454 family inner membrane protein	0.251	0.029	0.116
	tfaS	Pseudogene, CPS-53 (KpLE1) prophage	0.465	0.036	0.078
	yhbS	GNAT family putative N-acetyltransferase	0.286	0.016	0.054
	gfcC	Putative O-antigen capsule production periplasmic protein	0.283	0.014	0.048
	ybfL	Pseudogene, DDE domain transposase family	0.168	0.000	0.000
	yohC	Yip1 family inner membrane protein	0.205	0.000	0.000
	ypfN	Putative membrane protein, UPF0370 family	0.518	0.000	0.000
	yphA	DoxX family inner membrane protein	0.158	0.000	0.000
II	*cstA*	Carbon starvation protein involved in peptide utilization	0.186	0.152	0.815
	dsdX	D-serine transporter	0.342	0.075	0.219
	rhtB	Homoserine, homoserine lactone and S-methyl-methionine efflux pump	0.381	0.031	0.082
	Mtr	Tryptophan transporter of high affinity	0.197	0.000	0.000
III	*mhpD*	2-keto-4-pentenoate hydratase	0.154	0.053	0.345
	umuD	Translesion error-prone DNA polymerase V subunit	0.166	0.047	0.284
	mraZ	DNA-binding transcriptional repressor MraZ	0.149	0.038	0.254
	folX	Dihydroneopterin triphosphate 2'-epimerase	0.259	0.059	0.227
	ybaK	Cys-tRNA(Pro)/Cys-tRNA(Cys) deacylase	0.218	0.043	0.197
	wecF	4-acetamido-4,6-dideoxy-D-galactose transferase	0.306	0.060	0.196
	fimF	Minor component of type 1 fimbriae	0.277	0.051	0.186
	gloB	Hydroxyacylglutathione hydrolase	0.217	0.039	0.181
	rluF	23S rRNA pseudouridine (2604) synthase	0.164	0.028	0.170
	rpnD	Recombination-promoting nuclease RpnD, N-terminal fragment	0.254	0.043	0.170
	dcrB	Periplasmic bacteriophage sensitivity protein DcrB	0.185	0.031	0.169
	sfsB	*malPQ* operon transcriptional activator	0.205	0.029	0.141
	Nac	Nitrogen assimilation regulon transcriptional regulator	0.225	0.032	0.141
	glrR	DNA-binding transcriptional activator	0.249	0.034	0.138
	moeB	Molybdopterin synthase sulfurylase	0.219	0.030	0.135
	Fis	Global DNA-binding transcriptional dual regulator	0.361	0.044	0.122
	napF	Ferredoxin-type protein	0.290	0.034	0.119
	fsaA	Fructose-6-phosphate aldolase 1	0.197	0.021	0.107
	dgoA	2-oxo-3-deoxygalactonate 6-phosphate aldolase	0.374	0.039	0.105
	phoB	Response regulator in two-component regulatory system with PhoR	0.336	0.033	0.099
	bluR	Repressor of blue light-responsive genes	0.466	0.041	0.089
	chbG	Chito-oligosaccharide deacetylase	0.385	0.019	0.048
	yfjU	CP4-57 prophage	0.310	0.000	0.000
	ygiN	Quinol monooxygenase	0.495	0.000	0.000
	ecnB	Entericidin B membrane lipoprotein	0.180	0.000	0.000
	ubiJ	Conserved protein involved in ubiquinone-8 biosynthesis	NA	NA	NA
	esrE	EsrE small RNA	NA	NA	NA
	rhaD	Rhamnulose-1-phosphate aldolase	NA	NA	NA
Others[b]	MG1655[c]	Wild type	0.408	0.037	0.092
	BW25113[c]	Wild type	0.349	0.028	0.081
	ybeR	Uncharacterized protein	0.161	0.000	0.000
	yhaH	DUF805 family inner membrane protein	0.209	0.000	0.000
	yhjX	Pyruvate-inducible inner membrane protein, putative transporter	0.258	0.000	0.000
	yieP	Putative transcriptional regulator	0.179	0.000	0.000
	actP	Acetate transporter	0.185	0.042	0.227
	btsT	Inducible pyruvate transporter	0.195	0.000	0.000
	cstAybdD[d]	Double knockout strain of *cstA* and *ybdD*	0.326	0.112	0.343
	cstAbtsT[d]	Double knockout strain of *cstA* and *btsT*	0.342	0.148	0.434

[a]52 genes were selected from Tn-seq and tested their growth in bioTek. NA : Not determined

because its KEIO strain doesn't exist.

[b]10 of strains were additionally tested their growth in bioTek.

[c]MG1655 and BW25113 are the strain name (not a gene name) that was used for control strain in the growth assay as a wild type.

[d]*cstAybdD* and *cstAbtsT* are the double knockout strains.

Table S3. Bacterial strains used in this study.

Strain	Genotype	References
MG1655	K-12 F$^-$λ$^-$ *ilvG*$^-$ *rfb-50 rph-1*	(1)
BW25113	Δ(*araB-D*)*567* Δ(*rhaD-B*)*568* Δ*lacZ4787*(::*rrnB-3*) *hsdR514 rph-1*	(2)
Δ '*gene_name*'	Δ(*araB-D*)*567* Δ(*rhaD-B*)*568* Δ*lacZ4787*(::*rrnB-3*) *hsdR514 rph-1* Δ '*gene_name*'	(2)

Table S4. Primers used in this study.

Primer Name	Primer sequence (5' to 3')
Transposon_confirm_F	GACGGGACGGCGGCTTTGTTGAATA
Random_primer_1	GTTCAGACGTGTGCTCTTCCGATCNNNNNNNNGCTGG
Random_primer_2	GTTCAGACGTGTGCTCTTCCGATCNNNNNNNNCCAGC
Random_primer_3	GTTCAGACGTGTGCTCTTCCGATCNNNNNNNNCTGGC
Random_primer_4	GTTCAGACGTGTGCTCTTCCGATCNNNNNNNNTGGCG
Specific_Tn_F	ACACTCTTTCCCTACACGACGCTCTTCCGATCTCCTGCAGGCATGCAAGCTTCAGGGTTGAG
Truseq_Adapter_5R	CAAGCAGAAGACGGCATACGAGATCACTGTGTGACTGGAGTTCAGACGTGTGCTCTTCC
Truseq_Adapter_6R	CAAGCAGAAGACGGCATACGAGATATTGGCGTGACTGGAGTTCAGACGTGTGCTCTTCC
Truseq_Adapter_12R	CAAGCAGAAGACGGCATACGAGATTACAAGGTGACTGGAGTTCAGACGTGTGCTCTTCC
Posi_2nd_F	AATGATACGGCGACCACCGAGATCTACACTCTTTCCCTACACGACGCTCTTCCGATCT
cstA_KO_F	TATAGAAACAAAATGTAACATCTCTATGGACACGCACACGGATAACAACTGTGTAGGCTGGAGCTGCTTC
ybdD_KO_R	TGCGTGCAGCGTTCAAAAAAGTGGAATCGTTTAAAGCCTGACGTACAAATCTGTCAAACATGAGAATTAA
yjiY_KO_F	TAGTTCACTCTGATAAGAACAAAGCCCCGCCGAAGCGGGGCTAAACACGGGTGTAGGCTGGAGCTGCTTC
yjiY_KO_R	ACCTAGAACGGCTTCGGCCAACTATTAATCAATACATGCCAGGTTTTACTCTGTCAAACATGAGAATTAA

REFERENCES

1. Jensen KF. 1993. The *Escherichia coli* K-12 "wild types" W3110 and MG1655 have an rph frameshift mutation that leads to pyrimidine starvation due to low *pyrE* expression levels. J Bacteriol 175:3401-7.
2. Baba T, Ara T, Hasegawa M, Takai Y, Okumura Y, Baba M, Datsenko KA, Tomita M, Wanner BL, Mori H. 2006. Construction of *Escherichia coli* K-12 in-frame, single-gene knockout mutants: the Keio collection. Mol Syst Biol 2:2006 0008.
